# Individual and Combined Effects of Commercial Glyphosate and Dicamba-Based Herbicide Formulations on Cytotoxicity, Oxidative Stress and Cell Death on Human Intestinal Caco-2 Cells

**DOI:** 10.3390/jox16050168

**Published:** 2026-09-04

**Authors:** Gisele de Paula Júlio Garcia, Carolina Silva Schiebel, Maiara Vicentini, Daniele Maria-Ferreira, Izonete Cristina Guiloski

**Affiliations:** 1 Faculdades Pequeno Príncipe, Av. Iguaçu, 333, Curitiba 80230-020, PR, Brazil; gisele.garcia@aluno.fpp.edu.br (G.d.P.J.G.); carolina.schiebel@aluno.fpp.edu.br (C.S.S.); daniele.ferreira@professor.fpp.edu.br (D.M.-F.); 2 Instituto de Pesquisa Pelé Pequeno Príncipe, Av. Silva Jardim, 1632, Água Verde, Curitiba 80250-060, PR, Brazil; 3 Instituto Carlos Chagas, Fundação Oswaldo Cruz, Rua Prof. Algacyr Munhoz Mader, 3775, Curitiba 80350-010, PR, Brazil; maiaravicentini@gmail.com

**Keywords:** pesticides, enterocytes, oxidative stress, inflammation, apoptosis

## Abstract

Pesticide contamination of food and environmental matrices represents a potential risk to human health. This study investigated the toxicological effects of commercial glyphosate and dicamba-based herbicide formulations, individually and in combination, on human intestinal epithelial Caco-2 cells. Cells were exposed to a concentration range of 0.1–10,000 mg/L for 24, 48, and 72 h to determine IC_50_ values. Subsequent assays were conducted using concentrations based on the lowest IC_50_ obtained and in the limits established by the Environmental Protection Agency for drinking water. After 48 h of exposure, glyphosate at the highest tested concentration, as well as co-exposure to glyphosate and dicamba, induced significant cytotoxicity, modulation of oxidative stress parameters, and alterations in antioxidant defenses, accompanied by increased rates of apoptosis and necrosis. Dicamba exposure alone also resulted in elevated apoptotic and necrotic cell populations. A reduction in N-acetyl-β-D-glucosaminidase activity was observed across most tested concentrations, suggesting impaired inflammatory response capacity. This work identified alterations in Caco-2 that impair cellular homeostasis by cytotoxicity, alterations in oxidative stress, antioxidant response, inflammation, apoptosis and necrosis. Future studies investigating inflammatory pathways, genetic damage, and assays with in vivo and in silico models are important to elucidate the mechanism of the cellular damage caused by exposure to these herbicides.

## 1. Introduction

Pesticides are synthetic chemical substances used mainly to control, eliminate or prevent the development of plants, insects, larvae, fungi, ticks and rodents in rural and urban environments [1,2]. The population increase and the need for greater food production have driven a greater use of pesticides in world agriculture, leading to the accumulation of these residues in food, water, and soil, causing environmental contamination and consequently damage to human health and that of other animals [3,4,5].

Brazil ranks first in the production and export of soybeans and sugarcane, with a current volume of 96.8 million tons and 46 million tons, respectively [6,7]. In corn production, Brazil is the third largest producer in the world (132.07 million tons), preceded by the USA (348.75 million tons) and China (277.20 million tons) [7]. These three products alone were responsible for 85% of the annual area harvested throughout the country in 2017 [8,9] and corresponded to the destination of more than 70% of the pesticides sold in the country in 2022 [10], with glyphosate alone responsible for approximately 58% of consumption [11].

Glyphosate is used on a large scale and is among the most widely used herbicides in the world. It has been associated with DNA damage, carcinogenesis, mutagenesis, and effects on reproduction [12]. However, some crops have developed resistance to this substance, necessitating the development of alternatives for use in combination with other herbicides, such as dicamba [13,14]. Dicamba is a water-soluble substance and can move in the environment due to its volatility. Although it is not classified as a carcinogen, several studies have associated dicamba with lymphomas, thyroid cell carcinoma, colon cancer, multiple myeloma, non-Hodgkin’s lymphoma, and prostate cancer [15,16]. Regulatory agencies establish health-based benchmarks to assess the potential risks associated with exposure to individual chemical substances. However, a specific health-based benchmark for the combined exposure to glyphosate and dicamba has not been established. This is particularly relevant given the diversity of commercial formulations containing these active ingredients and the potential contribution of formulation components to their toxicity. The Drinking Water Equivalent Level (DWEL) is a health-based concentration in drinking water corresponding to a lifetime exposure level at which adverse non-carcinogenic health effects are not expected, assuming that exposure occurs entirely through drinking water. The DWEL values reported for glyphosate and dicamba are 70 mg/L and 18 mg/L, respectively [17].

The persistence and accumulation of these products in the environment may result in co-exposure that can be harmful to health, highlighting the need for continuous monitoring and investigation to mitigate potential harm [18]. Moreover, regulatory frameworks typically assess these substances individually, without adequately considering co-exposure or their potential cumulative toxicological effects on human health and the environment [19].

The gastrointestinal system is the first barrier between the food and water consumed and the body, constituting the front line of defense against the cytotoxic effects of pesticides. While nutrients are absorbed in the small intestine, toxic compounds can also be taken up through this organ [20,21]. Over the course of life, we consume a wide range of potentially harmful substances, and the involuntary ingestion of pesticides has been associated with disruption of intercellular junctions in mammalian enterocytes [22].

The Caco-2 cell line, derived from human colon adenocarcinoma, is used as an in vitro model of the human intestinal barrier, as it mimics the intestinal epithelial monolayer [23,24]. Caco-2 intestinal cells have been used to evaluate necrosis and oxidative stress on exposure to glyphosate-based herbicide [25], as well as oxidative stress and cell death induced by pesticides [26] and the uptake of herbicides across the apical membranes of intestinal cells [27].

Since there is no data in the literature on the toxicological effects of the co-exposure to glyphosate- and dicamba-based herbicides on the human intestinal cell line Caco-2, this study evaluated cytotoxicity, oxidative stress, inflammatory biomarkers, induction of apoptosis and necrosis, and changes in intestinal permeability to determine whether these herbicides could compromise the functionality of these cells.

## 2. Materials and Methods

### 2.1. Cell Culture

Caco-2 cells were acquired from the Rio de Janeiro Cell Bank (BCRJ; code 0059). Cells were expanded in 75 cm^3^ polypropylene flasks containing Dulbecco’s Modified Eagle’s Medium/F12 (DMEM/F12; Sigma-Aldrich, St. Louis, MO, USA), supplemented with 10% fetal bovine serum (FBS) and 1% antibiotics (10,000 IU mL^−1^ penicillin and 10 mg mL^−1^ streptomycin) of the prepared volume; the cells were maintained at 37 °C in a humidified incubator with 5% CO_2_. This culture medium was used to prepare the herbicide-containing solutions at the established concentrations. All assays were performed with at least three independent biological replicates.

### 2.2. Herbicides

Glyphosate-based herbicides (CAS number: 1071-83-6; Zapp QI 620^®^; Syngenta, Sao Paulo, Brazil; 620 g/L) and dicamba (CAS number: 1918-00-9; Atectra^®^; BASF SA, Sao Paulo, Brazil; 480 g/L) were used in this study. Glyphosate is supplied in the potassium-salt form, with the molecular formula C_3_H_7_NO_5_P·K and an acid equivalent of 500 g/L [28]. Dicamba is formulated as the 2-(2-aminoethoxy) ethanol salt of 3,6-dichloro-o-anisic acid (dicamba DGA salt) at a concentration of 708 g/L, equivalent to 480 g/L of free dicamba acid, with the molecular formula C_11_H_15_Cl_2_NO_5_ [29]. Additional formulation components, including surfactants, solvents, stabilizers, and anti-foaming agents, are also present. However, the public product labels do not necessarily disclose the individual chemical identities and concentrations of all formulation components, which may be collectively described as inert ingredients.

### 2.3. Cytotoxicity and IC_50_ Determination

To evaluate cytotoxicity, the PrestoBlue (PrestoBlue™ Cell Viability Reagent, Invitrogen, Carlsbad, CA, USA) assay was used. This method is based on the reduction of non-fluorescent blue resazurin to pink, fluorescent resorufin through cellular metabolic activity. The resulting fluorescence can be detected using absorbance-based plate readers [30].

The medium containing the pesticides was removed, then 90 μL of culture medium and 10 μL of the PrestoBlue™ reagent were added. The plate was incubated at 37 °C for 90 min. The reading was performed in a spectrophotometer at absorbance of 570 and 600 nm. The calculation of the oxidation/reduction of PrestoBlue™ was performed according to the manufacturer’s guidelines and normalized in relation to the control. The experiment was carried out in three independent biological replicates.

The cells were plated at a concentration of 2 × 10^4^ per well in 96-well plates. After 48 h, cells were exposed to dilution of the commercial herbicide formulations. The glyphosate- and dicamba-based products used in this study contain commercial formulation concentrations of 620 and 480 g/L of active ingredient, respectively, with glyphosate supplied in its potassium-salt formulation. Based on the concentrations provided by the manufacturers, the required volume of each formulation was calculated to obtain a final herbicide concentration of 100,000 mg/L for preparation of the stock solution in distilled water. Working solutions were subsequently prepared by diluting the stock solution in complete cell-culture medium consisting of 89% DMEM-F12, 10% FBS, and 1% P/S. All working solutions were prepared immediately before use and were not stored. Test concentrations were obtained using the equation C1 × V1 = C2 × V2, where C1 was the stock solution concentration (100,000 mg/L), V1 the volume of stock solution used for dilution, V2 the final preparation volume (3 mL), and C2 the target concentrations (0.1, 1, 10, 100, 1000, and 10,000 mg/L). Serial dilutions were performed from the highest to the lowest concentration. For preparation of the herbicide mixture, the same procedure was applied, with both herbicides diluted simultaneously.

The working concentrations used, expressed as active-ingredient equivalents, were tested to determine the IC_50_ values (50% inhibitory concentration) and thus determine the concentrations of the subsequent assays, according to OECD 129 [31]. For IC_50_ determination, exposure times of 24, 48 and 72 h were evaluated.

The IC_50_ value obtained after 72 h of exposure, which was the lowest value observed compared with the other exposure times, was used because a smaller amount of the substance was required to reduce cell viability by 50%, indicating greater cytotoxic potency. The experimental groups for the subsequent assays were defined (Table 1). Two additional comparative groups were established: one group with a concentration fivefold lower than the IC_50_ and another corresponding to the Drinking Water Equivalent Level (DWEL), according to EPA (2018) [32]. The control group consisted of cells maintained in culture medium without pesticide addition.

### 2.4. Oxidative Stress Analysis

The cells were plated at a concentration of 2 × 10^5^ cells/well in six-well plates and kept in an incubator for 48 h. Then, they were exposed to the herbicides glyphosate and dicamba at the concentrations defined above (Section 2.3). After 48 h of exposure, the cells were collected by trypsinization and then centrifuged at 900 rpm for 10 min. The supernatant was discarded, and the pellet was resuspended in 1000 μL of phosphate-buffered saline (PBS) to remove residual medium. The samples were centrifuged again under the same conditions, the supernatant was removed, and the pellet was resuspended in 500 μL of PBS.

The samples were then placed in a freezer at −30 °C for 30 min to promote cell lysis. After this period, they were transferred to microtubes and aliquoted for the quantification of total proteins, superoxide dismutase (SOD), glutathione peroxidase (GPx), glutathione S-transferase (GST), reduced glutathione concentration (GSH) and lipoperoxidation (LPO). The microtubes were stored at −80 °C until analysis.

For the quantification of total proteins, samples (10 μL) were added in a 96-well plate, in triplicate, followed by the addition of 250 μL of Bradford reagent (Sigma^®^). A standard curve was prepared with bovine serum albumin (BSA), at concentrations of 0; 0.125; 0.250; 0.500 and 1.0 mg BSA/mL. The reading was performed at a wavelength of 595 nm and the results were expressed in mg of protein/mL [33].

The analysis of SOD activity was performed according to the method proposed by Gao et al. (1998) [34]. First, 40 μL of the sample was added in microtubes, followed by 885 μL of buffer (Tris 1 M, EDTA 5 mM, pH 8.0) and 50 μL of pyrogallol 15 mM. Samples were incubated for 30 min protected from light. The reaction was stopped by adding 25 μL HCl 1N. Subsequently, 200 μL of each sample, in triplicate, was added in 96-well microplates. Absorbance was measured at 440 nm and the SOD activity was expressed in U/mg of protein.

GPx activity was measured according to the method of Paglia and Valentine (1967) [35]. The assay is based on the GPx-catalyzed oxidation of reduced glutathione (GSH) to oxidized glutathione (GSSG), coupled with the immediate reduction of GSSG back to GSH by exogenous glutathione reductase (GR), with concomitant NADPH consumption. In a 96-well microplate, 10 μL of sample was pipetted in triplicate. After this, 130 μL of the reaction solution 1 containing sodium azide at 3.1 mM, NADPH at 0.31 mM, GSH at 3.1 mM, glutathione reductase at 1.54 U/mL and sodium phosphate buffer (NaH_2_PO_4_, 0.1 M, pH 7.0) was added. After incubation for two minutes, 60 μL of solution 2 containing hydrogen peroxide (1.5 mM) and sodium phosphate buffer (NaH_2_PO_4_—0.1 M, pH 7.0) was added. The plate was incubated for five minutes protected from light. Then, the reading was performed at 340 nm every 30 s for five minutes. GPx enzymatic activity was expressed in nmol·min^−1^·mg^−1^ protein.

The activity of the GST enzyme was evaluated by the method described by Keen et al. (1976) [36]. First, 20 μL of each sample was added to 96-well microplates, after 180 μL of the reaction solution composed of CDNB (1-chloro-2,4-dinitrobenzene) 3 mM, GSH 3 mM and phosphate buffer (0.1 M, pH 6.5). The plate was incubated for five minutes. Then, the absorbance was read at a wavelength of 340 nm every 30 s for five minutes. The enzymatic activity GST was expressed in nmol/min/mg protein.

For GSH determination, the method proposed by Sedlak and Lindsay (1968) [37] was used, which measures free sulfhydryl levels, including those present in proteins and free cysteine, in addition to GSH. First, 100 μL of the samples was placed in microtubes, and 25 μL of 50% trichloroacetic acid was added. Samples were vortexed and centrifuged at 10,000× *g* for 10 min at 4 °C. Then, 25 μL of each sample was added to the 96-well microplates, followed by 230 μL of Tris-base Buffer (400 mM; pH 8.9) and 20 μL of DTNB (5.5′-dithio-bis-2-nitrobenzoic acid) at 2.5 mM (prepared in 25% methanol with Tris-base buffer 400 mM, pH 8.9). GSH concentration was determined by comparison with a standard GSH curve (0, 2.5, 5, 10, 20, 40 and 80 μg/mL). A single absorbance reading was performed at 415 nm, and the results were expressed in μg of GSH/mg protein.

Lipoperoxidation was assessed by malondialdehyde (MDA) quantification according to the method of Heath and Packer (1968) [38]. First, 70 μL of each sample was mixed with 300 μL of a solution containing 20% trichloroacetic acid and 0.5% thiobarbituric acid in microtubes. The microtubes were incubated at 95 °C for 30 min. Then, the reaction was stopped by placing the samples on ice for 10 min. Samples were added to 96-well microplates in triplicate. Absorbance was measured at 532 and 600 nm, and the results were expressed as nmol of MDA/mg protein.

### 2.5. Analysis of Inflammation Markers

Cells were plated at a concentration of 2 × 10^5^ cells per well in six-well plates and kept in an incubator for 48 h. Then, they were exposed to the herbicides glyphosate and dicamba at the concentrations previously defined (Section 2.3). After 48 h of exposure, the cells were collected by trypsinization and then centrifuged at 900 rpm for 10 min. The supernatant was discarded, and the pellet was resuspended in 1000 μL of sodium phosphate buffer (NaH_2_PO_4_, 0.1 M, pH 7.0), to remove residual medium. The samples were centrifuged again under the same conditions, the supernatant was removed, and the pellet was resuspended in 200 μL of 0.5% HTAB (trimethyl ammonium hexadecyl bromide) diluted in PBS. The samples were stored in a freezer at −30 °C for 30 min to promote cell lysis. After lysis, the samples were aliquoted for the analysis of N-acetyl-β-D-glucosaminidase (NAG) and myeloperoxidase (MPO) activities.

For the MPO activity, 30 μL of sample was added to 96-well microplates along with 100 μL of phosphate buffer (0.08 M), 85 μL of phosphate buffer (0.22 M) and 15 μL of H_2_O_2_ (0.017%). The reaction was initiated by adding 20 μL of 3.3′,5.5′-tetramethylbenzidine (TMB) and 3 min later it was stopped with 30 μL of sodium acetate (1.46 M, pH 3.0). Absorbance was measured at 620 nm and the results were expressed as optical density (OD) [39].

NAG activity was determined according to the method proposed by Bailey (1988) [40]. The NAG solution (3.44 mM) was prepared using 4-nitrophenyl n-acetyl-β-D-glucosaminide (≥99% purity, Sigma Aldrich, St. Louis, MO, USA). In a 96-well microplate, 25 μL of sample was added along with 25 μL of NAG solution and 100 μL of citrate buffer (50 mM; pH 4.5). The microplate was incubated for 60 min, after which 100 μL of glycine buffer (200 mM; pH 10.4) was added. The reading was performed by spectrophotometry at 405 nm, and the results were expressed as optical density (OD).

### 2.6. Evaluation of the Potential Inducer of Apoptosis and Necrosis

Apoptosis induction was evaluated using a phosphatidylserine externalization assay with Annexin V-FITC labeling (Annexin V conjugates for Apoptosis Detection, catalog number 13199; Invitrogen™). Late apoptosis and loss of membrane integrity were assessed using 7-AAD (7-Aminoactinomycin D; catalog number A1310; Invitrogen™). Cells were initially seeded at a density of 2 × 10^5^ cells per well in six-well plates and incubated for 48 h. Subsequently, cells were exposed to glyphosate- and dicamba-based herbicides according to the experimental groups described in Section 2.3.

After 48 h of exposure, the culture medium was collected and transferred to labeled tubes corresponding to each experimental group. The wells were washed with 500 μL of PBS, and the washing solution was added to the respective tubes. Cells were then detached using 500 μL of trypsin. After neutralization with 500 μL of culture medium, the cell suspension was also transferred to the corresponding tubes. For the control condition, non-exposed cells were cultured under the same conditions as the experimental groups and subjected to the same cell detachment procedure.

Samples were centrifuged at 900 rpm for 10 min, and the cells were resuspended in 200 μL of labeling buffer (Binding Buffer/PBS, 1:10). Subsequently, 5 μL of 7-AAD and 3 μL of Annexin V-FITC were added to each sample. The tubes were incubated for 15 min at room temperature and protected from light. After incubation, 100 μL of labeling buffer was added to each tube.

Data acquisition was performed by collecting 10,000 events per sample using a FACS Canto II^®^ flow cytometer (BD^®^—Becton, Dickinson and Company, Franklin Lakes, NJ, USA), with Annexin V-FITC detected in the FITC channel and 7-AAD detected in the PerCP channel. Data were analyzed using Infinicyt™ Flow Cytometry Software version 1.6.0 (Cytognos S.L., Santa Marta de Tormes, Salamanca, Spain).

Compensation was performed using unstained cells and cells individually stained with FITC and PerCP fluorophores. Unstained cells were used to define basal fluorescence/autofluorescence and establish the negative fluorescence boundaries. Untreated cells stained with Annexin V-FITC and 7-AAD were used as biological controls.

The cell population of interest was first identified based on FSC-A and SSC-A parameters, with debris excluded from the analysis. Annexin V-FITC-positive cells were subsequently identified, and this population was further evaluated according to 7-AAD fluorescence. Annexin V+/7-AAD+ cells were classified as late apoptotic/advanced cell death, whereas Annexin V+/7-AAD− cells were classified as early apoptotic. Annexin V−/7-AAD+ cells, characterized by loss of membrane integrity, were considered compatible with necrotic cell death. Annexin V−/7-AAD− cells were considered viable. Gates were established using unstained cells as the negative control to define fluorescence thresholds and discriminate positive and negative populations.

### 2.7. Membrane Permeability

Intestinal permeability was assessed using cells exposed to DWEL-based reference concentration (group 1) and 1/5 of the IC_50_ (group 2), focusing on membrane and junctional damage at the lowest tested concentrations. Cells were seeded at a density of 2 × 10^4^ cells in Transwell plates (Sigma-Aldrich, CLS3460-48EA), consisting of 12-well transparent polystyrene inserts with polycarbonate membranes (0.4 µm pore size). Cultures were maintained at 37 °C in a humidified atmosphere with 5% CO_2_ for 21 days to allow differentiation into absorptive enterocytes, establishment of epithelial polarization, and formation of tight junctions. The culture medium was replaced twice weekly in both apical and basolateral compartments.

After differentiation, cells were exposed for 48 h to glyphosate and dicamba, individually and in combination (Section 2.3). After the exposure, the contents of the wells were removed, and both compartments were washed twice with PBS. After washing, PBS was then added to the wells (500 µL apical; 1500 µL basolateral) and incubated for 1 h. The apical compartment was subsequently replaced with 500 µL of Evans blue solution (170 µg/mL, 1% BSA), and translocation across the epithelial monolayer was monitored as an indicator of paracellular transport. Absorbance was measured at 630 nm at baseline, 30, 60, and 120 min. An Evans blue standard curve (0.244; 0.488; 0.976; 1.953; 3.906; 7.812; 15.625; 31.25; 65.5; 125 and 170 µg/mL) was used for quantification. Cell permeability results were expressed in μg of Evans Blue/hour/cm^2^ [41].

### 2.8. Statistical Analysis

Statistical analysis was performed using GraphPad Prism^®^ 5.0 software. For each experimental condition, three biological replicates were performed, with three technical replicates for each biological replicate. The results were expressed as mean ± standard error of the mean. For IC_50_ determination, cytotoxicity data were tabulated, log-transformed, and normalized, followed by non-linear concentration-response regression analysis. For cytotoxicity, oxidative stress, inflammation, apoptosis assays and intestinal permeability, data normality was assessed using the Shapiro–Wilk test. Then, data were analyzed by one-way ANOVA followed by Tukey’s post-test. In the graphs, statistical differences were represented by letters, with identical letters indicating no significant difference and distinct letters indicating significance (*p* < 0.05).

For the integration of results, Permutational Multivariate Analysis of Variance (PERMANOVA) was performed, applying 999 permutations. Principal Coordinate Analysis (PCoA) was also conducted to assess the general patterns, followed by correlation between the variables and the axes obtained from the PCoA. These multivariate analyses were conducted in R environment 4.4.2.

## 3. Results

### 3.1. Calculation of IC_50_

**Figure 1:**
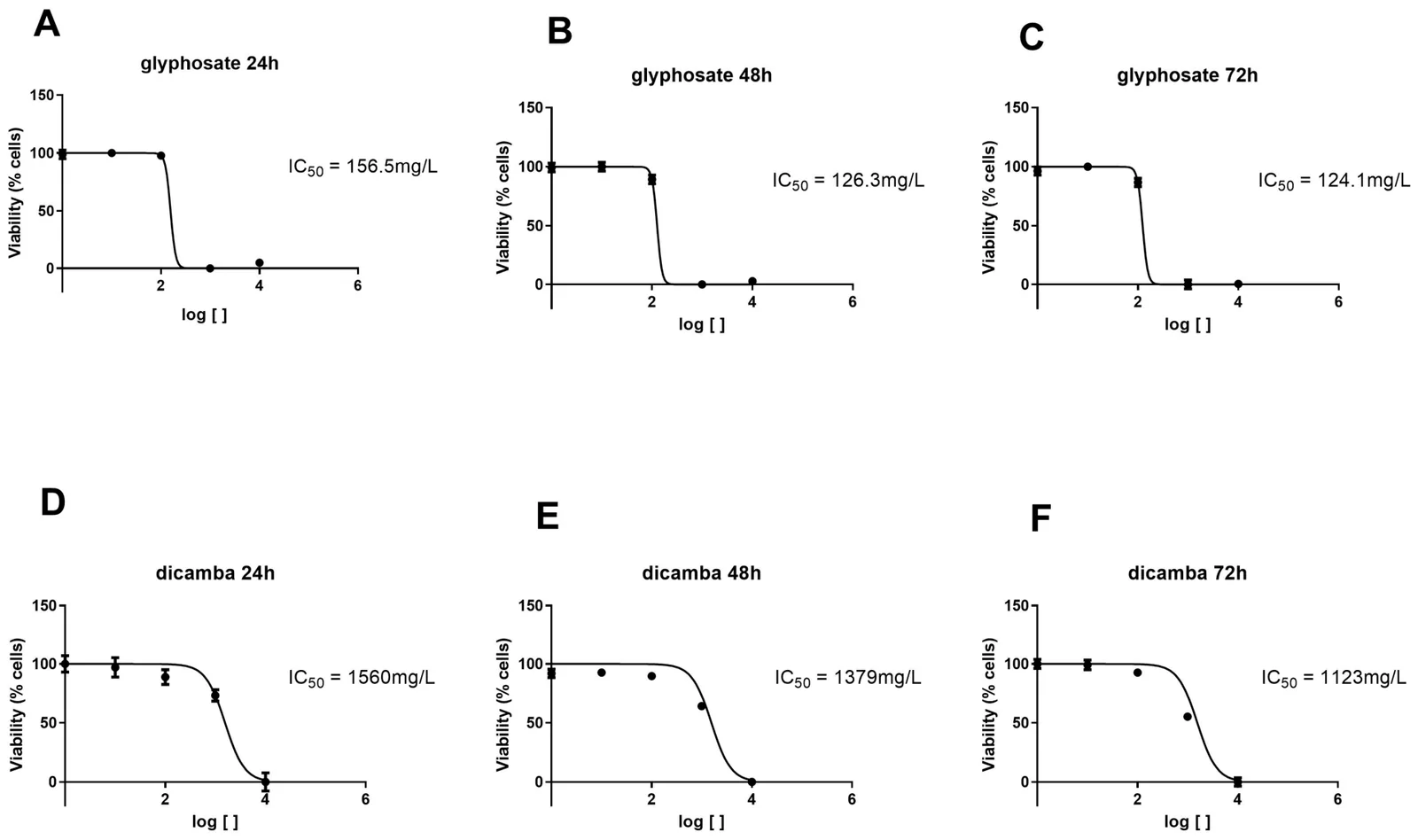
Response curve to the concentration of exposure to glyphosate and dicamba herbicides in Caco-2 cells showing the IC_50_ with data in percentage and concentrations expressed in log. 95% confidence interval: (**A**) 24-h glyphosate exposure; (**B**) 48-h glyphosate exposure; (**C**) 72-h glyphosate exposure; (**D**) 24-h dicamba exposure; (**E**) 48-h dicamba exposure; (**F**) 72-h dicamba exposure.

The determination of the IC_50_ occurred at 24 h, 48 h, and 72 h. For glyphosate, the IC_50_ was 156.5 mg/L at 24 h, 126.3 mg/L at 48 h, and 124.1 mg/L at 72 h (Figure 1A, Figure 1B and Figure 1C, respectively). For dicamba, the IC_50_ was 1560 mg/L at 24 h, 1379 mg/L at 48 h, and 1123 mg/L at 72 h (Figure 1D, Figure 1E and Figure 1F, respectively).

### 3.2. Cytotoxicity

**Figure 2:**
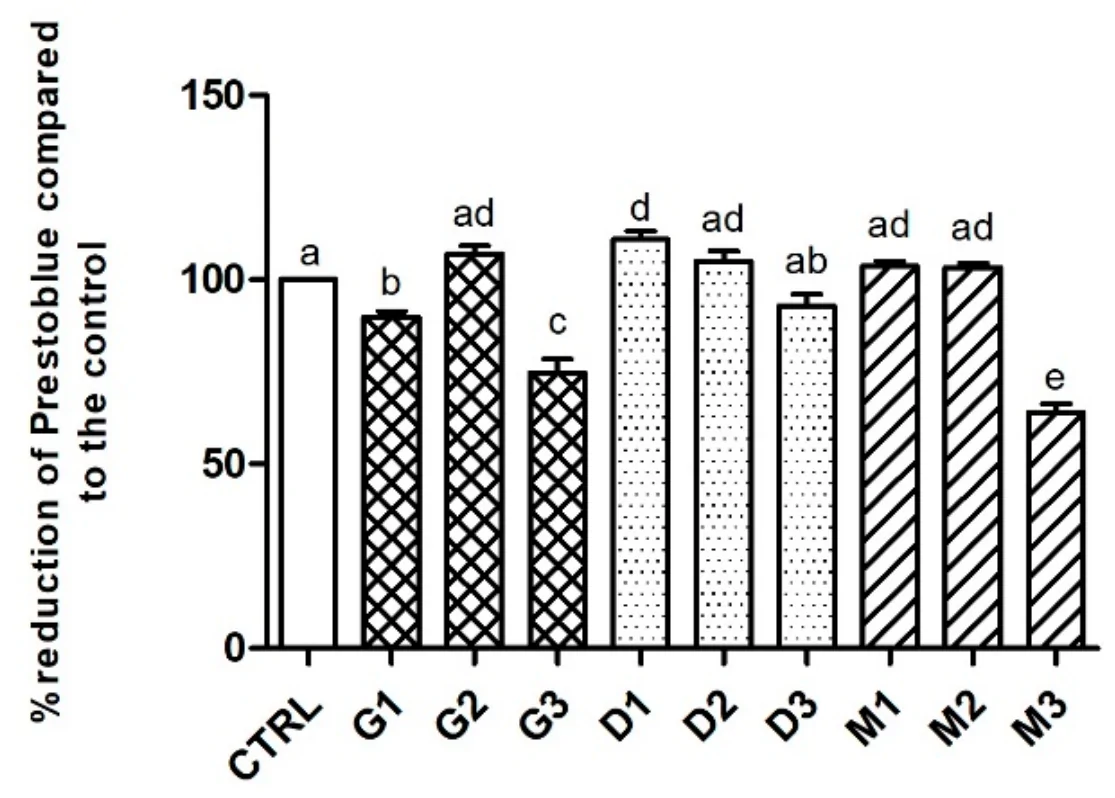
Cytotoxicity in Caco-2 cell line, exposed to glyphosate and dicamba-based herbicides both separately and in combination for 48 h. CTRL = control, G1 = glyphosate 70 mg/L, G2 = glyphosate 25 mg/L, G3 = glyphosate 124 mg/L, D1 = dicamba 18 mg/L, D2 = dicamba 224 mg/L, D3 = dicamba 1123 mg/L, M1 = co-exposure of glyphosate 70 mg/L and dicamba 18 mg/L, M2 = co-exposure of glyphosate 25 mg/L and dicamba 224 mg/L, M3 = co-exposure of glyphosate 124 mg/L and dicamba 1123 mg/L. Different letters (a, b, c, d, e) indicate statistically significant differences among groups (*p* < 0.05; one-way ANOVA followed by Tukey’s test). Three biological replicates were analyzed per group, with each biological replicate performed in technical triplicate (*n* = 3).

After the IC_50_ was determined, tests were conducted with the study groups (Table 1; Section 2.3) for a 48-h exposure time to assess cytotoxicity. One-way ANOVA showed an increased cytotoxicity in the Caco-2 cells after glyphosate exposure in groups G1, G3, and M3. However, in D1 there was a reduction in cytotoxicity compared to the control [F_(9,90)_ = 63.00; *p* < 0.05] Figure 2).

### 3.3. Induction of Oxidative Stress

**Figure 3:**
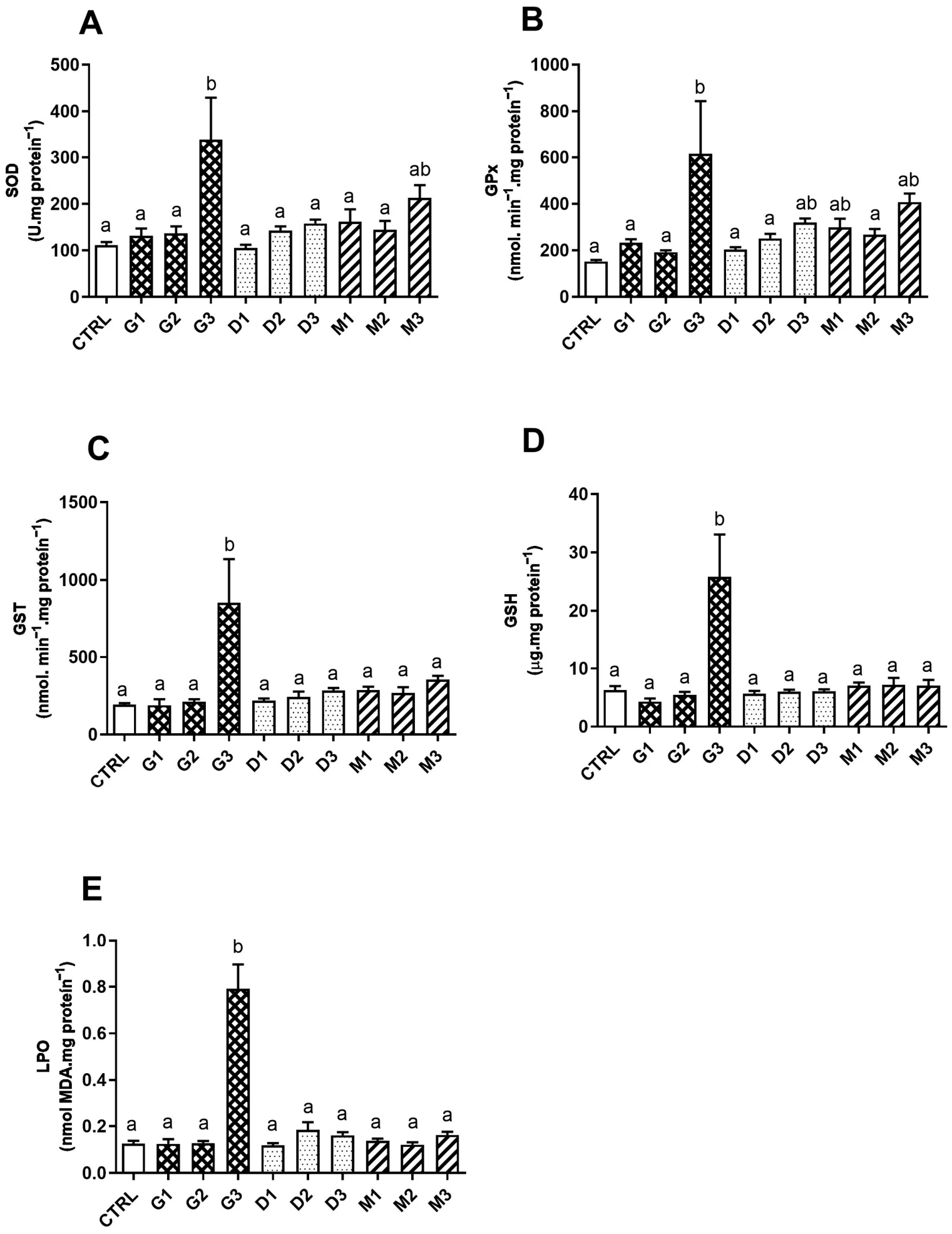
Biomarkers of oxidative stress in Caco-2 cells after 48 h of exposure to glyphosate and dicamba-based herbicides both separately and in combination. (**A**). SOD = superoxide dismutase; (**B**). GPx = glutathione peroxidase; (**C**). GST = glutathione S-transferase; (**D**). GSH = reduced glutathione; (**E**). LPO = lipoperoxidation. CTRL = control, G1 = glyphosate 70 mg/L, G2 = glyphosate 25 mg/L, G3 = glyphosate 124 mg/L, D1 = dicamba 18 mg/L, D2 = dicamba 224 mg/L, D3 = dicamba 1123 mg/L, M1 = co-exposure of glyphosate 70 mg/L and dicamba 18 mg/L, M2 = co-exposure of glyphosate 25 mg/L and dicamba 224 mg/L, M3 = co-exposure of glyphosate 124 mg/L and dicamba 1123 mg/L Three biological replicates were analyzed per group, with each biological replicate performed in technical triplicate (*n* = 3). The results are expressed as mean ± standard error of the mean. Different letters (a, b) indicate statistically significant differences between groups (*p* < 0.05). One-way ANOVA followed by Tukey’s post-test.

After 48 h of exposure of Caco-2 cells to the pesticides glyphosate and dicamba, a significant increase in antioxidant enzyme activity was observed for the experimental group G3, compared to the control and the other groups analyzed. In SOD activity (Figure 3A), G3 showed a statistical difference from the control and from group M3 [F_(9,78)_ = 4.832; *p* < 0.05]. In GPx activity (Figure 3B), G3 presented a statistical difference from the control and from groups G1, G2, D1, D2, and M2 [F_(9,75)_ = 4.210; *p* < 0.05]. The GST enzyme (Figure 3C) also showed statistical difference in G3, increasing its activity [F_(9,74)_ = 5.567; *p* < 0.05]. Regarding GSH concentration [Figure 3D; F_(9,66)_ = 7.553; *p* < 0.05] and LPO (Figure 3E; [F_(9,78)_ = 32.22; *p* < 0.05], G3 also demonstrated a significant increase compared to the control.

### 3.4. Analysis of Inflammation Markers

**Figure 4:**
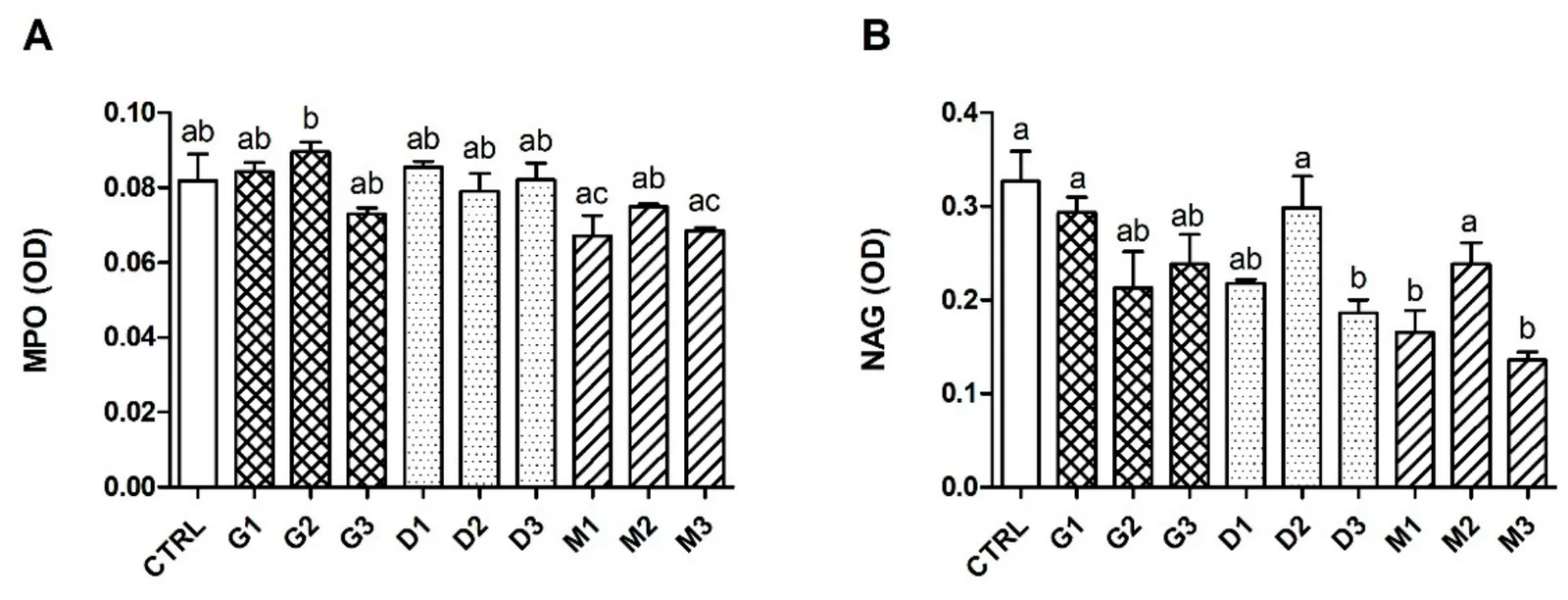
Myeloperoxidase (MPO) and N-Acetyl-glucosaminidase (NAG) activities after exposure to glyphosate and dicamba-based herbicides both separately and in combination, at 48 h. (**A**). MPO activity after 48 h of exposure and (**B**). NAG activity after 48 h of exposure. CTRL = control, G1 = glyphosate 70 mg/L, G2 = glyphosate 25 mg/L, G3 = glyphosate 124 mg/L, D1 = dicamba 18 mg/L, D2 = dicamba 224 mg/L, D3 = dicamba 1123 mg/L, M1 = co-exposure of glyphosate 70 mg/L and dicamba 18 mg/L, M2 = co-exposure of glyphosate 25 mg/L and dicamba 224 mg/L, M3 = co-exposure of glyphosate 124 mg/L and dicamba 1123 mg/L. Three biological replicates were analyzed per group, with each biological replicate performed in technical triplicate (*n* = 3). The results are expressed as mean ± standard error of the mean. Different letters (a, b, c) indicate statistically significant differences between groups (*p* < 0.05). One-way ANOVA followed by Tukey’s post-test.

In the analysis of MPO activity after 48 h of exposure to Caco-2 cells, it was observed that the groups did not differ statistically from the control (*p* > 0.05); however, there was a statistical difference between the groups G2, which showed an increase compared to M1 and M3 [F_(9,75)_ = 3.265; *p* < 0.05] (Figure 4A). Regarding the NAG analysis, a reduction in its activity was observed in relation to the control [F_(9,80)_ = 5.924; *p* < 0.05] for the groups D3, M1, and M3 (*p* < 0.05) (Figure 4B).

### 3.5. Analysis of the Inductive Potential of Apoptosis and Necrosis

**Figure 5:**
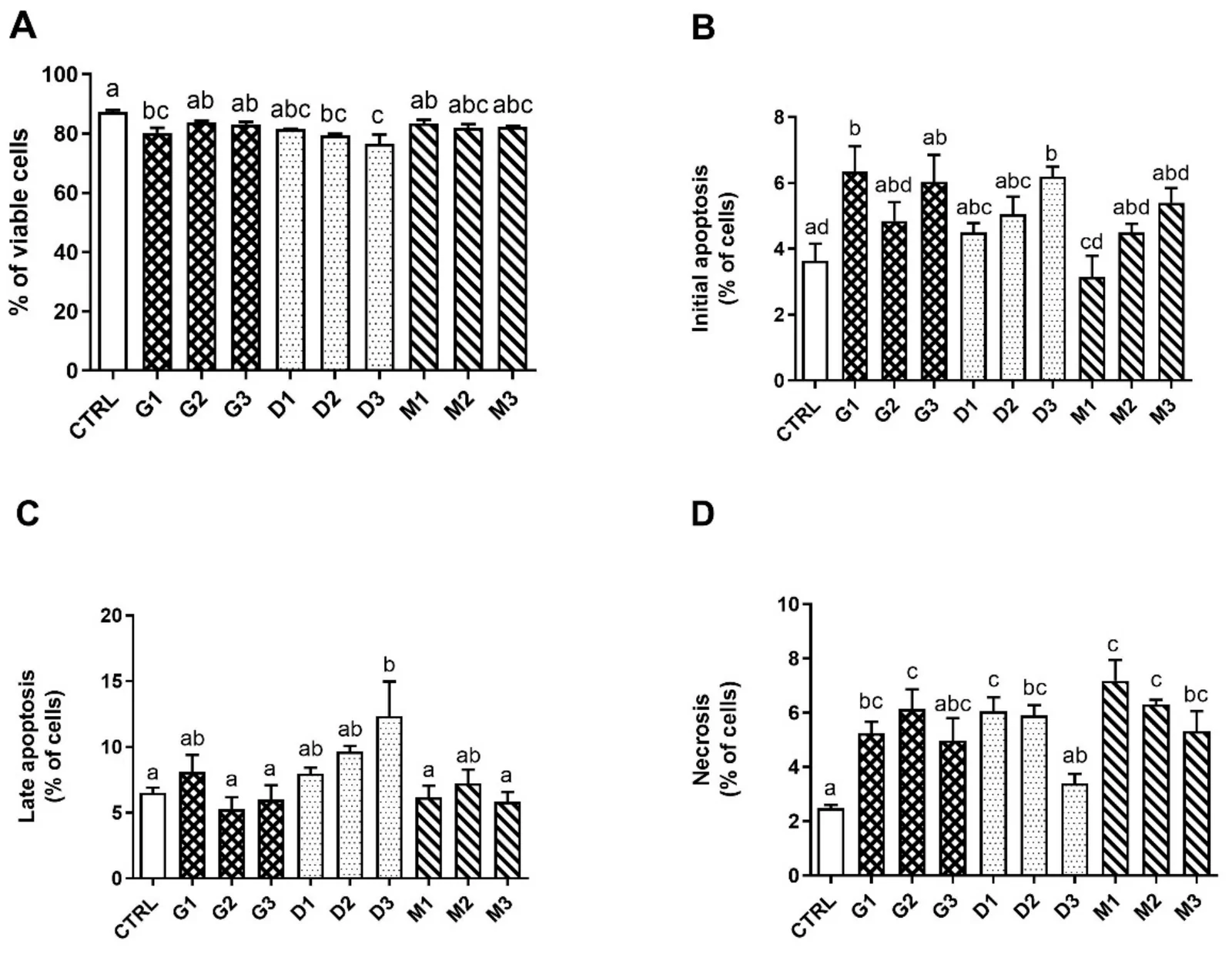
Assessment of apoptosis and necrosis in Caco-2 cells after exposure to glyphosate and dicamba-based herbicides both separately and in combination over 48 h. (**A**). Percentage of viable cells, negatively marked with annexin V and 7-AAD. (**B**). Percentage of cells in early apoptosis, marked only with annexin V. (**C**). Percentage of cells in late apoptosis, marked with annexin V and 7-AAD. (**D**). Percentage of cells in necrosis, marked only with 7-AAD. CTRL = control, G1 = glyphosate 70 mg/L, G2 = glyphosate 25 mg/L, G3 = glyphosate 124 mg/L, D1 = dicamba 18 mg/L, D2 = dicamba 224 mg/L, D3 = dicamba 1123 mg/L, M1 = co-exposure of glyphosate 70 mg/L and dicamba 18 mg/L, M2 = co-exposure of glyphosate 25 mg/L and dicamba 224 mg/L, M3 = co-exposure of glyphosate 124 mg/L and dicamba 1123 mg/L Three biological replicates were analyzed per group, with each biological replicate performed in technical triplicate (*n* = 3). The results are expressed as mean ± standard error of the mean. Different letters (a, b, c, d) indicate statistically significant differences between groups (*p* < 0.05). One-way ANOVA followed by Tukey’s post-test.

In the analysis of the potential to induce apoptosis and necrosis, after 48 h the herbicides glyphosate and dicamba showed a statistical difference compared to the control (Figure 5 and Figure S1). There was a reduction in the number of viable cells in the groups G1, D2, and D3 compared to the control (Figure 5A). Group D3 also differed from groups G2, G3, and M1 [F_(9,79)_ = 4.819; *p* < 0.05], demonstrating a reduction in viable cells.

The results demonstrated that G1 and D3 showed an increase in early apoptotic cells compared to the control (Figure 5B). Additionally, group M1 differed statistically from groups G1, G3, and D3 [F_(9,80)_ = 3.866; *p* < 0.05], which exhibited an increase in early apoptotic cells.

Regarding late apoptosis, group D3 showed a statistically significant difference from the control, G2, G3, M1, and M3 groups [F_(9,79)_ = 3.552; *p* < 0.05] (Figure 5C), increasing the number of cells in that condition. There was a statistically significant increase in necrotic cells for all groups [F_(9,80)_ = 6.413; *p* < 0.05]. This effect was not observed only in groups G3 and D3 (Figure 5D).

### 3.6. Membrane Permeability

The herbicides dicamba and glyphosate, tested individually or in combination, did not affect intestinal permeability in Caco-2 cells after 48 h of exposure (Figure S2; *p* > 0.05).

### 3.7. Multivariate Analysis

**Figure 6:**
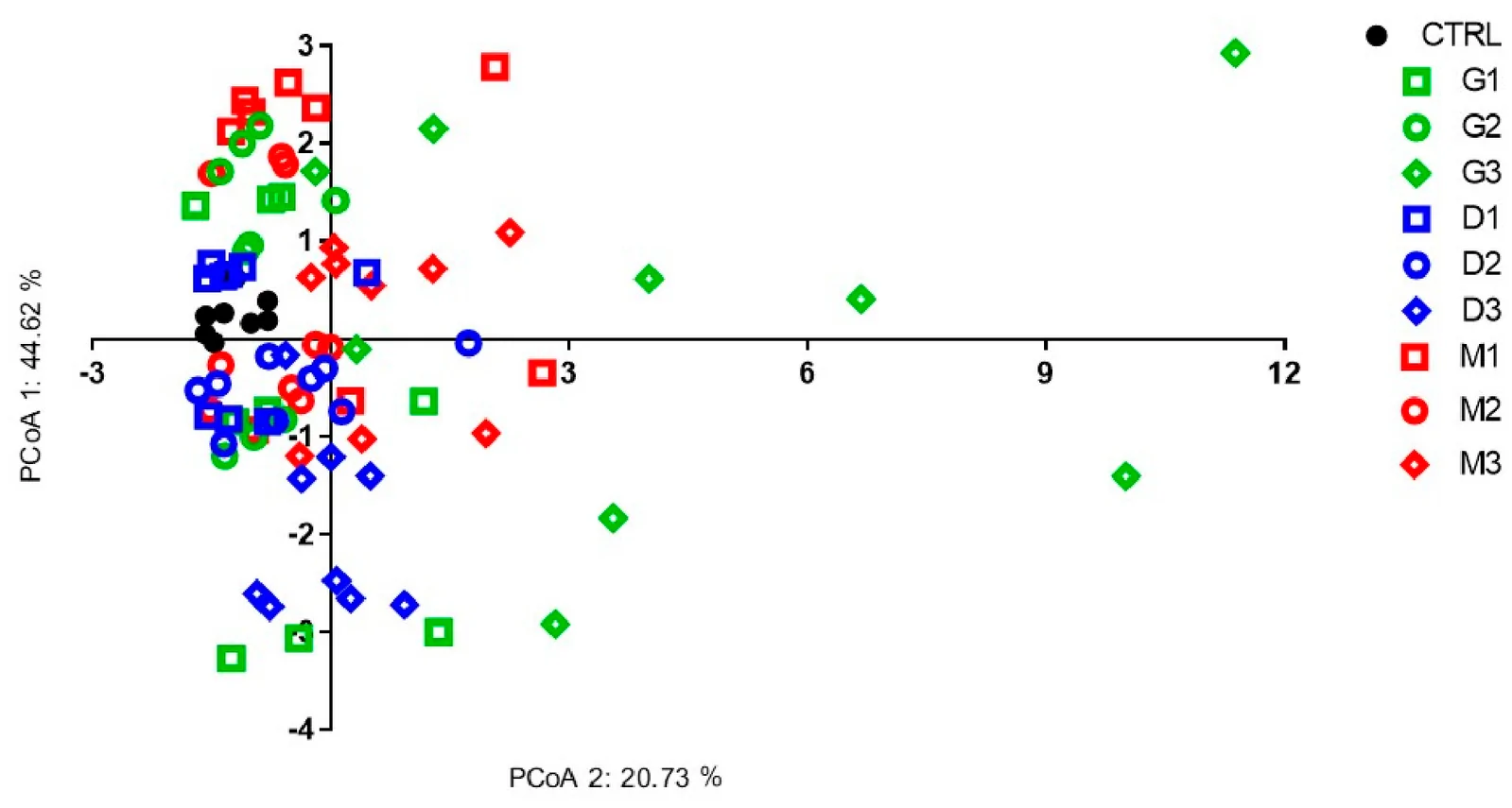
Analysis of Principal Coordinates (PCoA) in Caco-2 lineage cells, exposed to glyphosate and dicamba-based herbicides both separately and in combination for 48 h. CTRL = control, G1 = glyphosate 70 mg/L, G2 = glyphosate 25 mg/L, G3 = glyphosate 124 mg/L, D1 = dicamba 18 mg/L, D2 = dicamba 224 mg/L, D3 = dicamba 1123 mg/L, M1 = co-exposure of glyphosate 70 mg/L and dicamba 18 mg/L, M2 = co-exposure of glyphosate 25 mg/L and dicamba 224 mg/L, M3 = co-exposure of glyphosate 124 mg/L and dicamba 1123 mg/L.

PERMANOVA demonstrated a significant difference when combining data on cytotoxicity, NAG, and cell death markers (early apoptosis, late apoptosis, and necrosis). Based on this difference, a PCoA was performed to ordain the data and better visualize these differences (F = 7.724; R2 = 0.467; *p* = 0.001). Two axes of the PCoA explained 65.35% of all the data (44.62% for axis 1 and 20.73% for axis 2), as shown in Figure 6. It is possible to see that the control is concentrated in the upper left quadrant, while the treatment with G3 is in the right quadrants (both lower and upper) and D3 is in the lower quadrants (both right and left).

The variables related to these axes and their respective correlations are presented in Table S1. The biomarkers of oxidative stress (SOD, GPx, GST, GSH, and LPO), myeloperoxidase, and the number of cells in late apoptosis showed no correlation with any of the axes and therefore were not represented in the table. Cytotoxicity and NAG correlated negatively with axis 1. On the other hand, viable cells, apoptosis, and necrosis correlated with axis 2, with only apoptosis being negatively correlated.

## 4. Discussion

The use of pesticides has increased in recent years, and thus the exposure to these products has also risen. The gastrointestinal tract is a major route of entry when exposure occurs through contaminated water and food. Therefore, there is a risk to food safety and human health [18,42]. Chronic exposure to pesticides may impair digestive function by alter intestinal microbiota, disrupting gut–brain axis interactions, and inducing changes in permeability, as well as immunological, metabolic, and biochemical disturbances [43]. In this context, the present study evaluated two commercial formulations of herbicides used in agriculture and their effects on human intestinal Caco-2 cells. To determine the concentrations that would be used in the experiments, the IC_50_ of these herbicides was calculated.

The IC_50_ found for glyphosate was 124.1 mg/L and for dicamba it was 1123 mg/L at the longest exposure time (72 h) in Caco-2 cells. These values are equivalent to the concentrations of the herbicides in the commercial products. Based on these results, it can be observed that an approximately 10 times higher concentration of dicamba is needed to cause 50% mortality of the exposed cells compared to glyphosate, highlighting a greater cytotoxicity of glyphosate. This finding was confirmed in the 48-h cytotoxicity assay, in which glyphosate was cytotoxic at the highest concentration tested (G3; 124 mg/L), resulting in reduced cellular metabolic activity compared to the control. The G1 group (70 mg/L) also showed a similar effect, although to a lesser extent. Notably, the cytotoxicity observed following exposure to glyphosate in the commercial formulation (G1) at the DWEL-based concentration highlights the potential sensitivity of intestinal cells to this exposure level. However, the DWEL is a health-based reference value intended to estimate an acceptable level of exposure through drinking water and should not be directly interpreted as a threshold for cellular toxicity or as an indicator of human health risk. Therefore, the present findings should be interpreted with caution, as the in vitro concentrations used in this study do not directly represent human exposure levels. Nevertheless, the observed cellular effects at a DWEL-based concentration warrant further investigation, particularly considering the potential for exposure to pesticide residues through drinking water and other environmental sources [18,44,45].

Economic and political interests relax the tolerance limits allowed for pesticides. This can be observed in relation to glyphosate, for example, whose permitted concentration in water in Brazil is five thousand times higher (500 µg L^−1^) compared to the limits set by the European Union (0.1 µg L^−1^) [46]. Exposure to pesticide residues, which occur in mixtures in the environment, affects not only rural workers or residents of agricultural regions but all consumers [47].

Regarding the co-exposure, the M1 group showed a response comparable to the of control, despite the higher cytotoxicity observed in the isolated glyphosate (G1). The co-exposure at the highest concentration (M3) exhibited marked cytotoxicity, similar to the response observed in G3. These results indicate that there is a different pattern of response according to the concentration of the pesticide. The herbicide co-exposure evaluated in this study resulted in differential toxicological responses, which may reflect the combined effects of the active ingredients and other components present in the commercial formulations [48].

Toxicity assessment has shown variable effects depending on the formulation analyzed. Mesnage and collaborators reported that a commercial glyphosate formulation containing adjuvants and other constituents, was more toxic than the active ingredient alone, both in vitro (ToxTracker system using mammalian stem cells) and in vivo (Sprague-Dawley rats) [25]. Corroborating these results, the study by Ferguson and collaborators evaluated the toxic effects of glyphosate and dicamba in isolation, using the MTT assay in HepG2 hepatocarcinoma cells and immortalized fibroblasts, and also observed that commercial formulations exhibited greater toxicity than the isolated compounds [49]. Together with our findings, these studies highlight the importance of considering the composition of commercial formulations when interpreting the toxicity of glyphosate- and dicamba-based products. Because the present study evaluated commercial formulations, the observed effects cannot be attributed exclusively to the active ingredients, and the potential contribution of co-formulants should be considered.

One of the key mechanisms related to cytotoxicity is the excessive production of reactive oxygen species (ROS) combined with an insufficient antioxidant response, which can damage proteins, lipids, and DNA, consequently activating cell death via apoptosis and/or necrosis [50]. In the present study, the highest concentration of glyphosate (G3) induced the antioxidant system, evidenced by increased activities of SOD, GPx and GST, along with elevated GSH concentration, as well as induced lipoperoxidation. The antioxidant system works to balance the action of free radicals and ROS, aiming to inhibit or reduce their harmful effects on the cell. During cellular respiration, superoxide anions (O_2_^−^) are converted by SOD into hydrogen peroxide (H_2_O_2_). Subsequently, GPx along with GSH reduces H_2_O_2_ to less reactive compounds. GSH also serves as a reservoir and transporter of cysteine, participates in the detoxification of lipid peroxidation products, and acts as a reducing agent and enzymatic cofactor. In addition, GST contributes to cellular defense by facilitating the detoxification of metabolites generated under oxidative stress [51]. Together, these components perform complementary roles in the antioxidant defense system, exerting a combined effect to maintain cellular homeostasis.

In this study, although the antioxidant system was activated, it was not sufficient to prevent oxidative damage, as evidenced by membrane lipid oxidation. Excessive accumulation of free radicals promotes the oxidation of polyunsaturated fatty acid chains, leading to destabilization of the plasma membrane. This process compromises essential functions such as selective permeability and transport, and may also result in damage to proteins and DNA [52].

Lower concentrations of glyphosate did not alter the antioxidant system. Similarly, Silva et al. [26] reported no changes in antioxidant markers in Caco-2 cells exposed to low concentrations of isolated glyphosate. Neither the isolated dicamba groups nor the co-exposure groups showed changes in the antioxidant system of Caco-2 cells. The induction of oxidative stress is one of the disruptions associated with the toxic effects of glyphosate [53]. Although the G3 group exhibited oxidative stress, this effect was not observed in M3, suggesting a potential opposite effect of dicamba on glyphosate-induced oxidative damage.

To evaluate possible inflammatory effects caused by the herbicides glyphosate and dicamba, the markers myeloperoxidase (MPO) and N-acetyl-β-D-glucosaminidase (NAG) were analyzed. MPO is an enzyme released by activated neutrophils and acts as a marker of inflammation and cellular injury. MPO, when increased, acts in the catalysis of H_2_O_2_ and chloride ion (Cl^−^), which in turn produces hypochlorite (ClO^−^), becoming hypochlorous acid (HOCl). This compound when released can cause damage to biological molecules and inflammation [54]. Although the G3 group showed indications of increased oxidative activity, neither this group nor the others exhibited significant changes in MPO. The literature reports that organophosphate pesticides, such as malathion and malaoxon, can modulate myeloperoxidase activity [55].

NAG is a lysosomal enzyme widely recognized as a sensitive, non-specific biomarker of inflammation and tissue damage [56]. A significant reduction in NAG activity was observed in the D3, M1, and M3 groups, indicating a marked alteration in lysosomal function and cellular homeostasis after pesticide exposure. Since the tested concentrations correspond to levels considered safe by the EPA [57], the modulation of this biomarker raises concerns regarding their potential biological effects.

Although no inflammatory effect of glyphosate alone was observed in this work, another study found that glyphosate and its metabolite AMPA had pro-inflammatory potential in human neuroblastoma SH-SY5Y cells. In these cells, there was a significant increase in the gene expression of Interleukin 6 and tumor necrosis factor α [50].

In addition to oxidative stress and pro-inflammatory effects, cell death by apoptosis and necrosis was also observed. Cell death can occur through apoptosis or necrosis depending on the action pathway, being a physiological process of cellular regulation responsible for the balance necessary for cell renewal [58]. In the intestinal epithelium, cell death by apoptosis maintains homeostasis. However, when this process is inadequate, it leads to inflammatory conditions, resulting in acute and chronic injuries and imbalance of the intestinal barrier [59].

Oxidative stress-induced damage can increase cell death through both necrosis and late apoptosis. In this study, however, a predominance of necrosis was observed across all exposure groups, including those treated with individual herbicides and in the co-exposure (G1, G2, D1, D2, M1, M2, and M3). Consistent with these findings, Clair et al. [60] reported that a commercial formulation of glyphosate induced necrosis in Leydig, Sertoli, and germ cells following 1 to 48 h of exposure. Regarding dicamba, no reports of necrosis in cultured cells were found in the literature. However, an in vivo study using *Cyprinus carpio* L. fish as an experimental model showed that dicamba induced hepatocellular degeneration, vacuolization of hepatocytes, and infiltration of mononuclear cells [61].

In the present study, early apoptosis was observed in G1 and D3, while late apoptosis was detected in D3. Similarly, apoptotic induction has been reported in peripheral blood mononuclear cells (PBMCs) exposed to glyphosate at concentrations ranging of 0.01 to 5 mM. This response was characterized by increased membrane permeability accompanied by activation of caspases 8, 9, and 3, leading to chromatin condensation and apoptosis via both intrinsic and extrinsic pathways [62]. The herbicide 2,4-dichlorophenoxyacetic acid (2,4-D) in its commercial formulation (Dedalo Elite^®^) also caused an increase both early and late apoptosis rates in CHO-K1 cells (Chinese hamster ovary fibroblasts) [63]. Moreover, dicamba, whether applied alone or in combination with 2,4-D and mecoprop (MCPP), increased the number of apoptotic embryonic rat cells impairing embryonic development [64].

The observed results of necrosis and apoptosis at concentrations considered safe by the EPA (D1 = 18 mg/L, G1 = 70 mg/L, and M1 = D1 + G1) are of concern, as they were associated with reduced metabolic activity in intestinal cells, potentially compromising cellular homeostasis and the integrity of the intestinal barrier.

The intestinal barrier is composed of multiple interconnected components that regulate its permeability. The balance among dietary factors, microbiota, mucus layer, epithelial cells, and tight junction complexes is essential for maintaining intestinal microenvironment homeostasis, thereby providing both a physical and biochemical barrier against pathogens, toxins, and allergens [21]. Intestinal permeability was assessed using DWEL-based reference concentration in water and one-fifth of the IC_50_, in order to determine whether tight junction disruption and/or loss of membrane integrity occurred even at the lowest concentrations evaluated.

At the concentrations evaluated in this study, the herbicides glyphosate and dicamba did not alter permeability in Caco-2 cells. However, it has been reported that Caco-2 cell line forms particularly stronger tight junctions, conferring greater resistance compared to the small intestine in vivo [23]. In contrast, a study using glyphosate at concentrations greater than or equal to 10 mg/mL (equivalent to 10,000 mg/L) in Caco-2 and IEC-18 cells demonstrated a reduction in transepithelial electrical resistance (TEER) and an increase in permeability, alongside cytoskeletal disruption in both cell lines [65], clearly demonstrating intestinal barrier breakdown at high doses.

Another study demonstrated that both pure glyphosate and its commercial formulation Roundup at low concentrations also did not alter expression of key cell barrier proteins (Claudin 11, occludin, ZO-1, and connexin 43, 46, and 50) in Sertoli cells and germ cells in vivo (juvenile Sprague-Dawley rats) [66]. However, when the same cell type was evaluated in vitro (Sertoli cells), a decrease in transepithelial electrical resistance was observed, indicating impaired blood–testis barrier integrity, despite the absence of changes in the expression of membrane proteins such as: claudin 11, occludin, and ZO-1 [67]. This dysregulation impairs the function of the intestinal tight junctions, reducing their selectivity for the controlled passage of ions, minerals, water, and immune cells, as well as for the entry of pathogenic bacteria [23].

Finally, the present study demonstrated that glyphosate in commercial formulation, both alone and in co-exposure with dicamba significantly reduced cellular metabolism even at DWEL-based reference concentration. However, induction of the antioxidant response in the context of increased oxidative stress was only significant in Caco-2 cells exposed to isolated glyphosate at the highest concentration, whereas changes in cell viability and apoptosis were mainly observed in the D3 group. These findings are further supported by the PCoA analysis, in which these groups were clearly separated from the control group.

### Study Limitations

Despite these findings, limitations should be considered when interpreting the results. Although Caco-2 cells constitute a relevant in vitro model for investigating the potential effects of herbicides on intestinal cells, they do not fully reproduce the complexity of the human intestinal environment. In addition, the 48 h exposure period and the concentrations tested do not necessarily reflect real human exposure conditions. Moreover, the limited number of studies involving dicamba restricts direct comparisons with the existing literature. This study was conducted using commercial formulations; therefore, it is not possible to determine whether the observed effects were caused exclusively by the active ingredient, by the adjuvants present in the formulations, or by a combination of both. Thus, complementary investigations are needed to evaluate intestinal monolayers and other relevant cell types, as well as to examine the effects of these herbicides—individually and in combination—on gut microbiota activity. Additional analyses of inflammatory pathways in Caco-2 cells are also required, particularly to clarify the origin of the effects observed in the NAG assay and the oxidative stress response induced by the glyphosate-based herbicide.

## 5. Conclusions

This study investigated the effects of combined exposure to commercial formulations of the herbicides glyphosate and dicamba in Caco-2 cells. The results showed that co-exposure to these formulations altered parameters associated with cytotoxicity, oxidative stress, inflammatory responses, necrosis, and apoptosis, indicating disturbances in cellular homeostasis. Necrosis was observed in most experimental groups, although changes in oxidative stress markers and antioxidant responses were not consistently associated with these effects, suggesting that mechanisms beyond oxidative imbalance may contribute to the observed toxicity. Overall, these findings highlight the importance of considering co-exposure to commercial herbicide formulations when assessing their potential effects on intestinal cells and support further investigation of the molecular mechanisms underlying their combined toxicity and their possible implications for intestinal health.

## Figures and Tables

**Table 1:** Experimental group design and description.

Group	Herbicide Concentration
Control	0 mg/L
G1	70 mg/L glyphosate (DWEL)
G2	25 mg/L glyphosate (1/5 of the IC_50_ after 72 h)
G3	124 mg/L glyphosate (IC_50_ after 72 h)
D1	18 mg/L dicamba (DWEL)
D2	224 mg/L dicamba (1/5 of the IC_50_ after 72 h)
D3	1123 mg/L dicamba (IC_50_ after 72 h)
M1 (G1 + D1)	(70 mg/L glyphosate + 18 mg/L dicamba)—(DWEL)
M2 (G2 + D2)	(25 mg/L glyphosate + 224 mg/L dicamba)—(1/5 of the IC_50_ after 72 h)
M3 (G3 + D3)	(124 mg/L glyphosate + 1123 mg/L dicamba)—(IC_50_ after 72 h)

Note: DWEL—equivalent level allowed in drinking water [32]; glyphosate DWEL (G1) = 70 mg/L; dicamba DWEL (D1) = 18 mg/L; M1 = DWEL of G1 + DWEL of D1; IC_50_—Inhibitory concentration at 50%; 1/5 of the glyphosate IC_50_ (G2) = 25 mg/L; 1/5 of the dicamba IC_50_ (D2) = 224 mg/L; M2 = 1/5 IC_50_ of G2 + 1/5 IC_50_ of D2; glyphosate IC_50_ (G3) = 124 mg/L; dicamba IC_50_ (D3) = 1123 mg/L; M3 = IC_50_ of G3 + IC_50_ of D3.

## Data Availability

The original contributions presented in this study are included in the article/Supplementary Materials. Further inquiries can be directed to the corresponding author.

## Supplementary Materials

The following supporting information can be downloaded at: https://www.mdpi.com/article/10.3390/jox16050168/s1, Figure S1: Dot plot diagram of Caco-2 cells exposed to glyphosate and dicamba—based herbicides both separately and in combination for 48 h; Figure S2: Assessment of permeability on Caco-2 cells after exposure to glyphosate and dicamba—based herbicides both separately and in combination for 48 h; Table S1: Variables related to axes and their respective correlations.
