# Supplementary material for: Individual and Combined Effects of Commercial Glyphosate and Dicamba-Based Herbicide Formulations on Cytotoxicity, Oxidative Stress and Cell Death on Human Intestinal Caco-2 Cells
Source: J Xenobiot. 2026 Sep 4;16(5):168. doi: 10.3390/jox16050168 (PMC13608743; doi:10.3390/jox16050168)
Supplement: Supplementary file 1 [file jox-16-00168-s001.zip › jox-4447977-supplementary.pdf]

Individual and Combined Effects of Commercial Glyphosate and Dicamba-Based Herbicide Formulations on Cytotoxicity, Oxidative Stress and Cell Death on Human Intestinal Caco-2 Cells

Gisele de Paula Júlio Garcia, Carolina Silva Schiebel, Maiara Vicentini , Daniele Maria-Ferreira and Izonete Cristina Guiloski

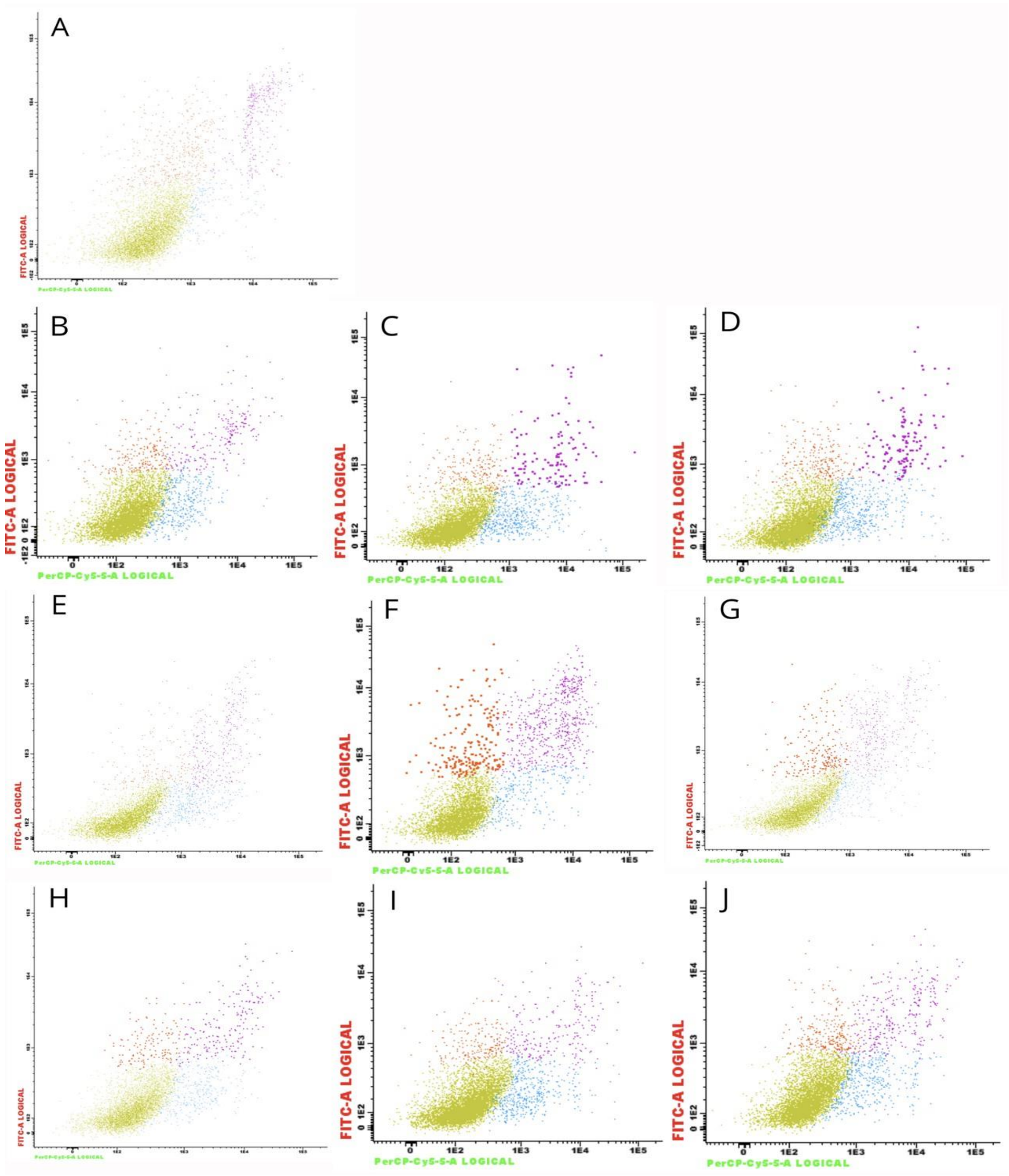

Figure S1. Dot plot diagram of Caco-2 cells exposed to glyphosate and dicamba - based herbicides both separately and in combination for 48h. (A) CTRL = no exposure, (B) G1 = glyphosate 70 mg/L, (C) G2 = glyphosate 25 mg/L, (D) G3 = glyphosate 124 mg/L, (E) D1 = dicamba 18 mg/L, (F) D2 = dicamba 224 mg/L, (G) D3 = dicamba 1123 mg/L, (H) M1 = glyphosate 70 mg/L and dicamba 18 mg/L, (I)) M2 = glyphosate 25 mg/L and dicamba 224 mg/L, (V) M3 = glyphosate 124 mg/L and dicamba 1123 mg/L. Viable cells are in the lower-left quadrant (Annexin IV and 7AAD negative),

cells in early apoptosis are in the lower-right quadrant (Annexin IV positive and 7AAD negative), cells in late apoptosis are in the upper-left quadrant (Annexin IV and 7AAD positive), and cells in necrosis are in the upper-right quadrant (Annexin IV negative and 7AAD positive).

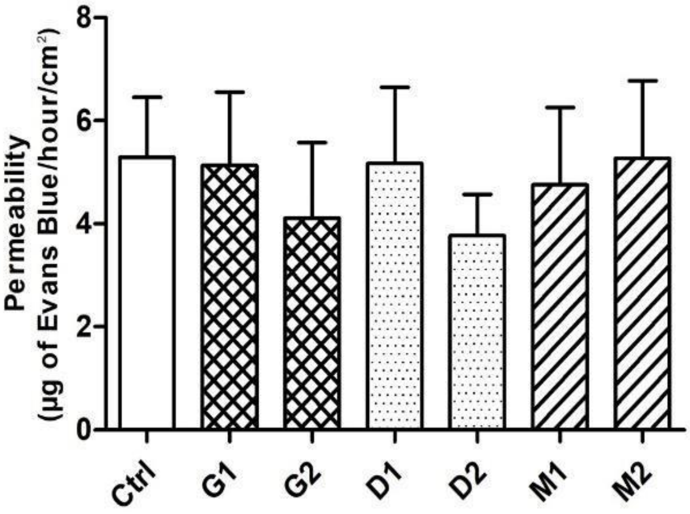

Figure S2. Assessment of permeability on Caco-2 cells after exposure to glyphosate and dicamba - based herbicides both separately and in combination for 48 hours. CTRL = control, G1 = glyphosate 70 mg/L, G2 = glyphosate 25 mg/L, D1 = dicamba 18 mg/L, D2 = dicamba 224 mg/L, M1 = co-exposure of glyphosate 70 mg/L and dicamba 18 mg/L, M2 = co-exposure of glyphosate 25 mg/L and dicamba 224 mg/L, M3 = co-exposure of glyphosate 124 mg/L and dicamba 1123 mg/L Three biological replicates were analyzed per group, with each biological replicate performed in technical triplicate (n = 3).

Table S1. Variables related to axes and their respective correlations.

Biomarker	Axis 1	Axis 2
Cytotoxicity	−0,4028	−0,1034
NAG	−0,1868	−0,1346
Viable Cells (Apoptosis Assay)	−0,0768	0,6866
Initial Apoptosis	0,2605	−0,7997
Necrosis	−0,0465	0,6386

Note: cytotoxicity and NAG correlated with axis 1, while viable cells, apoptosis, and necrosis correlated with axis 2. * $p<0.05$.
